# Acute Cerebellitis Associated With Anti-homer 3 Antibodies: A Rare Case Report and Literature Review

**DOI:** 10.3389/fneur.2022.837937

**Published:** 2022-02-18

**Authors:** Ailiang Miao, Chuanyong Yu, Yulei Sun, Lingling Wang, Jianqing Ge, Xiaoshan Wang

**Affiliations:** ^1^Department of Neurology, Affiliated Brain Hospital of Nanjing Medical University, Nanjing Medical University, Nanjing, China; ^2^Department of Video-Electroencephalpgram, Affiliated Brain Hospital of Nanjing Medical University, Nanjing Medical University, Nanjing, China; ^3^Department of Neurology, Affiliated Mingji Hospital of Nanjing Medical University, Nanjing Medical University, Nanjing, China

**Keywords:** cerebellitis, anti-Homer 3 antibody, head shaking, cerebellar syndrome, immunotherapeutic treatment

## Abstract

Acute cerebellitis associated with Homer-3 antibodies is very rare. Here we present a 20-year-old woman who suffered from uncontrollable head shaking quickly from side to side and an unsteady gait for 2 days after the cold. Antibodies were screened by cell-based assays. The indirect immunofluorescence technique results revealed anti-Homer-3 antibody titers of 1:3.2 in the CSF and 1:100 in the serum. The woman was obviously improved after antiviral and immunosuppression (immunoglobin, methylprednisolone and mycophenolate mofetil) treatment. Our report indicated immune-mediated causes should be considered in the acute cerebellitis. Immunotherapy can contribute to the improvement of cerebellar syndrome.

## Introduction

In 2013, Hoftberger et al. reported a 38-year-old man with anti-Homer 3 antibodies who presented with symptoms of acute encephalopathy including headache, nausea, vomiting, and confusion, and cerebellar syndrome (1). Furthermore, seven patients with subacute or insidious “idiopathic cerebellar ataxia,” not acute cerebellitis, were reported (2, 3). Acute cerebellitis associated with anti-Homer 3 antibodies is very rare. Here, we report a female with acute cerebellitis associated with anti-Homer 3 antibodies.

## Case Report

**Figure 1:**
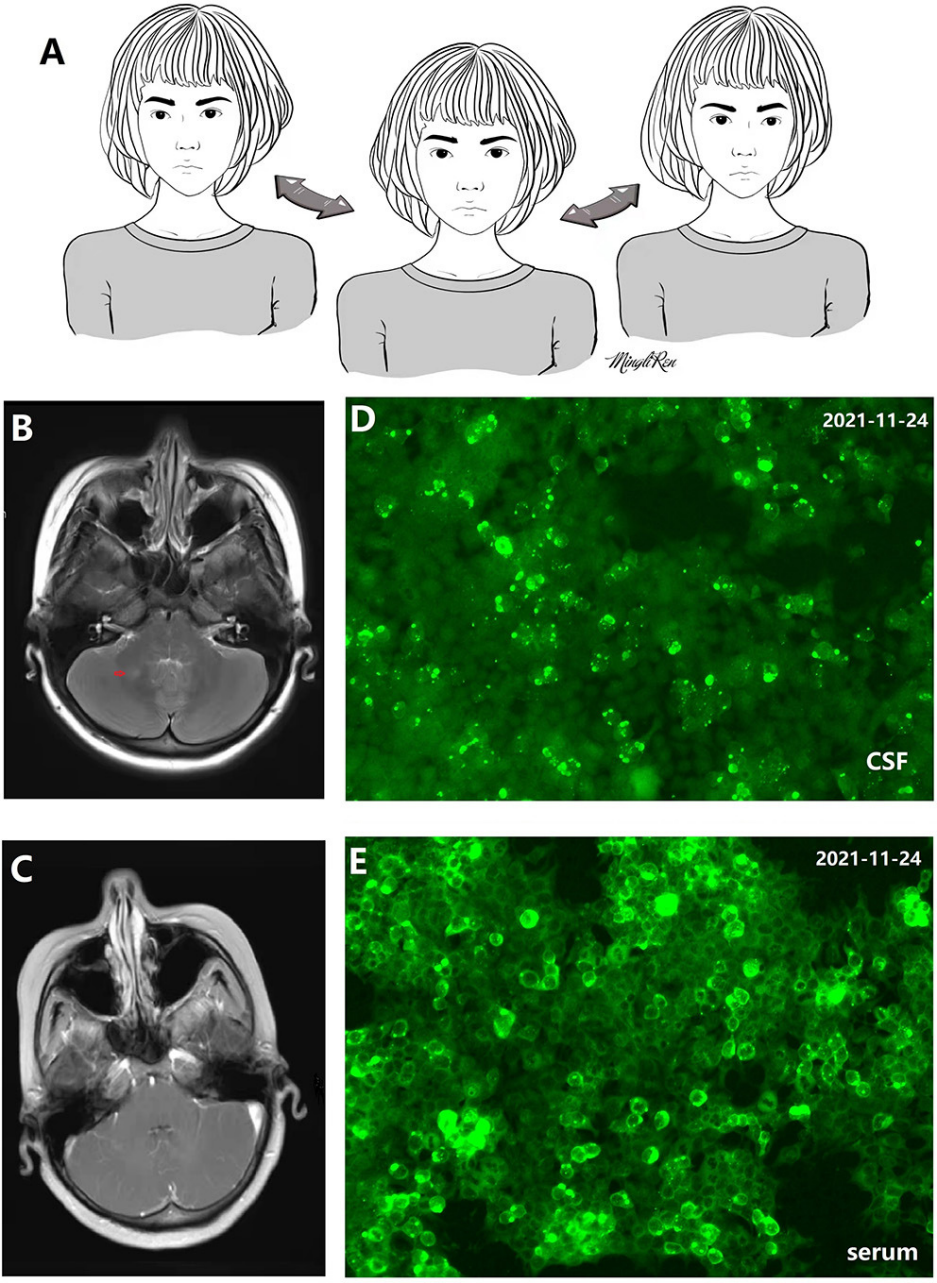
**(A)** 2D picture associated with Supplementary Video 1 showing uncontrollable and quick head shaking. **(B,C)** Increased signal in the right cerebellar hemisphere without enhancement. **(D)** Antibody in cerebrospinal fluid (CSF) recognizes Homer-3 antigens in fifixed HEK293 cells. Anti-Homer-3 antibody titers in CSF: 1:3.2. **(E)** Antibody in serum recognizes Homer-3 antigens in fifixed HEK293 cells. Anti-Homer-3 antibody titers in serum: 1:100.

**Figure 2:**
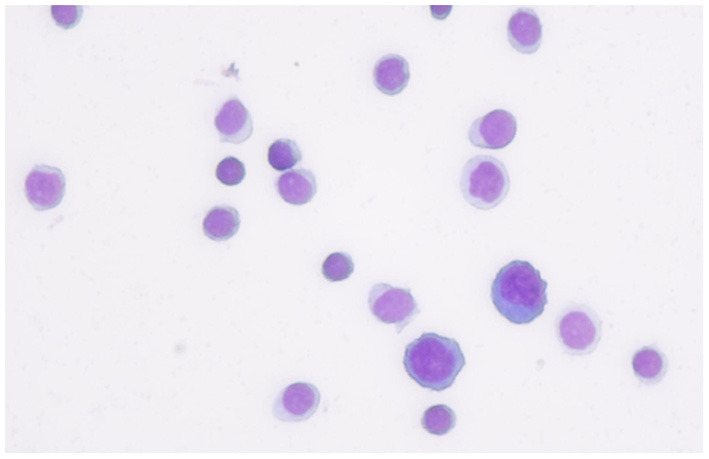
The first cerebrospinal fluid cytology from the patient showed that most of inflammatory cells were lymphocyte.

A 20-year-old woman suffered from uncontrollable head shaking twice quickly from side to side (Supplementary Video 1 and Figure 1A) for 2 days after the cold (Table 1). In other words, the head swayed twice quickly and slightly from side to side. The unsteady gait was also observed (Supplementary Video 2). The patient had a history of allergic rhinitis for 2 years. Neurological examination demonstrated bilateral horizontal nystagmus, moderate limb dysmetria, Romberg sign positivity and gait ataxia. The patient was admitted to Nanjing Brain Hospital. On day 1, brain magnetic resonance imaging (MRI) showed an increased signal in the right cerebellar hemisphere without enhancement (Figures 1B,C). On day 3, lumbar puncture was performed, and a pressure of 180 mmH_2_O, a WBC count of 139 × 10 ^6^ /L (Figure 2), and a protein level of 1.67 g/L were observed. The oligoclonal band was positive. On day 5, the indirect immunofluorescence technique (IIFT) results revealed anti-Homer-3 antibody titers of 1:3.2 in the CSF and 1:100 in the serum (Figures 1D,E). The gynecological sonography was normal. On day 4, mild diffuse waves were observed on electroencephalography. After 66 days of antiviral and immunosuppression (immunoglobin, methylprednisolone and mycophenolate mofetil) treatment, the woman was obviously improved (Supplementary Video 2, Table 1).

**Table 1:** The symptoms and treatment in the patient according to timeline.

**Symptoms and examination**	**Time**	**Treatment**
Intermittent headache; fatigue;	2021-10-7	Without treatment
Intermittent headache, neck pain; fatigue	2021-11-1	
Gait ataxia.	2021-11-16	
Head shaking uncontrollably from side to side (Supplementary Video 1 and Figure 1A); Gait ataxia (Supplementary Video 2).	2021-11-18	
The patient could not complete heel-knee-tibia test and finger-nose test stably, and presented moderate limb dysmetria, Romberg sign and horizontal nystagmus. The patient also could not walk in a straight line.	2021-11-20 Hospitalization	Ganciclovir for injection was administered and sustained by 0.375 g twice a day.
Increased signal in the right cerebellar hemisphere without enhancement	2021-11-20 Brain magnetic resonance imaging (MRI)	
WBC count:139 × 10 ^6^ /L (Figure 2); protein level:1.67 g/L.	2021-11-22 Lumbar puncture	
	2021-11-24	Anti-Homer-3 antibody titers of 1:3.2 in the CSF and 1:100 in the serum (Figure 1).
	2021-11-24	Methylprednisolone for injection was administered by 1,000 mg per day, and reduced by half every three days. Immunoglobin was administered by 25 g per day for 5 days (2 g per kilogram).
Gait ataxia, head shaking and horizontal nystagmus improved.	2021-11-30	
Gait ataxia, head shaking and horizontal nystagmus still improved.	2021-12-3	Mcophenolate mofetil was given and sustained by 0.5 g twice a day. Methylprednisolone was administered by 120 mg per day for 3 days.
The heel-knee-tibia test, finger-nose test and moderate limb dysmetria improved.	2021-12-6	Prednison was given by 60mg per day, and reduced by 5mg every two week.
Anti-Homer-3 antibody titers of 1:3.2 in the CSF and 1:100 in the serum. WBC count: 55 × 10 ^6^ /L; protein level: 0.91 g/L. Horizontal nystagmus was not observed. Gait ataxia and head shaking was still observed (Supplementary Video 2).	2021-12-9 Lumbar puncture	
Head shaking disappeared.	2021-12-15	
The improvement of gait ataxia was not remarkable. The patient still could not walk in a straight line.	2021-12-25	Another immunoglobin was administered by 25 g per day for 5 days (2 g per kilogram).
Normal	2022-1-5 Brain MRI	
WBC count: 29 × 10 ^6^ /L; protein level: 0.75g/L.	2022-1-13 Lumbar puncture	Ganciclovir and mcophenolate mofetil was administered and sustained.
Although the patient improved remarkably, mild gait ataxia and unbalance during walking in a straight line were still observed (Supplementary Video 2). The patient could complete both hands alternating movement test, heel-knee-tibia test and finger-nose stably, and did not present Romberg sign. Another lumbar puncture was not received by the patient.	2022-1-25 Hospital discharge	Acyclovir tablets were given by 0.4 g three times a day for two weeks. Mcophenolate mofetil was given and sustained by 0.5 g twice a day. Prednison was given by 50 mg daily, and reduced by 5 mg every two weeks.

## Discussion

We described a rare case of cerebellitis associated with Homer-3 antibodies. This patient was positive for the anti-Homer 3 antibody in the CSF and serum, but negative for anti-ATP1A3, ARHGAP26, ITPR1, Hu, Yo, Ri, CV2, Ma2, amphiphysin, Tr(DNER), Zic4, Ma1, GAD65, PKCγ, SOX1, NMDAR, AMPA1, AMPA2, GABAB, LG1, CASPR2, DPPX, lolON5, mGluR5, GlyRα1, GABAARα1, and GABAARβ3.

The Homer family includes Homer-1, Homer-2, and Homer-3, all of which have several isoforms as a result of alternative splicing (4). Homer proteins can be divided into the two structurally distinct groups of short and long Homer proteins. Short Homers include Homer-1A, Homer-2C, Homer-2D, Homer-3C and Homer-3D. Long Homer proteins include Homer-1B, Homer-1C, Homer-2A, Homer-2B, Homer-3A_xx_ and Homer-3B_xx_. The short~35 amino acid residue long coiled-coiled domain in the Homer N-terminals may be important for the folding of Homers themselves or involved in interacting with proteins. This short N-terminal coiled-coil domain is present in all Homer-3 proteins except for the Homer-3B. The short domain in Homer-3A is remarkable longer than that in Homer-3C and Homer-3D (5). Homer-3 and mGluR1 (metabotropic glutamate receptor) are expressed predominantly on Purkinje cell dendritic spines (6). Homer-3 is the scaffold protein between mGluR1 and inositol 1,4,5 triphosphate receptors, which regulate the post-synaptic calcium metabolism in Purkinje cells in response to mGLuR1 stimulation (7). Thus, the anti-Homer 3 antibodies might bind Homer-3A, Homer-3C and Homer-3D, especially Homer-3A disturbing the homer 3 function, which could contribute to cerebellar ataxia (1–3, 5). Cerebellar ataxia is also the most common symptom of anti-mGluR1 autoimmunity (8).

In 2007, Zuliani et al. reported a 65-year-old woman with Homer-3 antibodies presenting with subacute cerebellar ataxia. Although the patient received steroids, the cerebellar syndrome had not improved by the last follow-up (2). Guan et al. screened the serum and CSF samples of 750 patients with ‘idiopathic' cerebellar ataxia, and Homer-3 antibodies were detected in 6 patients. Interestingly, 2 patients had RBD, a hot cross bun sign, and dysautonomia, which may be considered diagnostic markers for multiple system atrophy of the cerebellar type (MSA-C) (3). Given that there is no effective treatment for MSA-C, immune-mediated cerebellar syndrome can be improved by immunotherapy (3). Homer-3 antibodies are even more rarer, and screening for antibodies in every patient with acute, subacute and insidious cerebellar syndrome is unrealistic. An interesting symptom, “head socking uncontrollably from side to side (Supplementary Video 1 and Figure 1A),” was observed in this patient with Homer-3 antibodies, which might be a characteristic of cerebellar syndrome with Homer-3 antibodies, and was not reported in the previous studies (1–3). Seasonable immunotherapy can contribute to the improvement of cerebellar syndrome, and delayed treatment might lead to unfavorable outcomes in patients with cerebellar ataxia (3). Figure 1A was depicted by a female patient with anti-N-methyl-d-aspartate receptor encephalitis in our hospital (9). Immunotherapeutic treatment was not delayed, and the patient had no residual problems (9).

In summary, we report a rare patient with cerebellitis with Homer-3 antibodies who improved after immunotherapeutic treatment. The symptom “head shaking” might lead to cerebellar syndrome associated with Homer-3 antibodies.

## Data Availability Statement

The original contributions presented in the study are included in the article/Supplementary Material, further inquiries can be directed to the corresponding author.

## Author Contributions

AM: drafting and revising the manuscript. XW: study concept or design and study supervision. CY, YS, LW, and JG: clinical work. Their contributions helped us to acquire clinical data. All authors contributed to the article and approved the submitted version.

## Funding

The work was supported by the Young Scientists Fund of the National Natural Science Foundation of China (Grant No. 81501126, http://npd.nsfc.gov.cn/), Science and Development Foundation of Nanjing Medical University (2014NJMU050), Young Medical Key Talents Foundation of Jiangsu Province (Grant No. QNRC2016053), and Training Project for Young Talents of Nanjing Brain Hospital. General project of Nanjing Municipal Health Commission (YKK21110).

## Conflict of Interest

The authors declare that the research was conducted in the absence of any commercial or financial relationships that could be construed as a potential conflict of interest.

## Publisher's Note

All claims expressed in this article are solely those of the authors and do not necessarily represent those of their affiliated organizations, or those of the publisher, the editors and the reviewers. Any product that may be evaluated in this article, or claim that may be made by its manufacturer, is not guaranteed or endorsed by the publisher.

## Supplementary Material

Supplementary Video 1 The patient presented head shaking twice uncontrollably and quickly from side to side, without body shaking.

Supplementary Video 2 The patient was administered antiviral and immunotherapies and improved.
